# Social media; a tool for retention?

**DOI:** 10.1186/1710-1492-10-S1-A4

**Published:** 2014-03-03

**Authors:** Claire R Unruh, Allan Becker

**Affiliations:** 1 Department of Pediatrics and Child Health, University of Manitoba, Winnipeg, Canada

## Background

Social media sites are becoming more and more popular within the healthcare industry. There is a huge potential in research for using different social media sites for retention and looking at their long term effects in a longitudinal study like the CHILD study – something not yet looked into at depth [1]. At present, most social media sites in research are involved in recruiting participants for studies. Social media provides an exploratory and informative environment for families involved in longitudinal studies. Researchers and study families can mutually share information – creating a web of connections through the internet. Such engagement may help in the retention of participants in a longitudinal study.

## Methods

### Facebook

Keep connections with study families in a timely fashion. Facebook is able to show what a research team does on a regular basis. Researchers can share information regarding recent and related publications. Because of the many connections of those signed in to Facebook, communities are built [2] and users become engaged. Facebook has the ability to poll the public on different issues and gives insights and data to group administration.

### Twitter

Keep connections with other health care professionals, researchers and members of the general public. There is a potential for collaboration. Talks directly to experts and “clients” and gives all the ability to connect to each other.

### Pinterest

Share allergy and asthma news with the public in one area. Pinterest is a collection of links to articles and websites about information surrounding research in pediatric allergy and asthma.

### Instagram

The sharing of photos. Researchers can share photos of the lab environment; including how to capture data and measurements. This aids in daily contact with the families of research studies. Benefits the families; allows the kids to see the testing environment and opens up links of communication.

The ability to link social media sites to each other offers the availability of information to be accessed in different means, furthering the webs of connection.

## Results

There are no results yet from the usage of social media for the retentions of a longitudinal cohort like CHILD. With more time and analysis, significant results are anticipated.

## Conclusions

There are limitations to using social media for a research study. There must be time allocated to updating each site as regularly as possible. The Internet is still not accessible to all despite being accessible to many. Information is only shared with those engaged in social media. In these cases, research studies must find other ways to ensure that the information is equally distributed. With the help of social media, longitudinal research studies are able to keep a presence in the daily lives of participating families. This helps to strengthen connections for the study and may ultimately help in retention. When looking at conducting a study, researchers should not shy away from social media sites.
